# Resistance to Thyroid Hormone Beta Due to *THRB* Mutation in a Patient Misdiagnosed With TSH-Secreting Pituitary Adenoma

**DOI:** 10.1210/jcemcr/luae140

**Published:** 2024-08-01

**Authors:** Wenjun Liao, Nipawan Waisayanand, Kanda Fanhchaksai, W Edward Visser, Marcel E Meima, Karn Wejaphikul

**Affiliations:** Department of Internal Medicine, Academic Center for Thyroid Diseases, Erasmus MC, 3015 CN, Rotterdam, the Netherlands; Department of Internal Medicine, Faculty of Medicine, Chiang Mai University, Chiang Mai, 50200, Thailand; Department of Pediatrics, Faculty of Medicine, Chiang Mai University, Chiang Mai, 50200, Thailand; Department of Internal Medicine, Academic Center for Thyroid Diseases, Erasmus MC, 3015 CN, Rotterdam, the Netherlands; Department of Internal Medicine, Academic Center for Thyroid Diseases, Erasmus MC, 3015 CN, Rotterdam, the Netherlands; Department of Pediatrics, Faculty of Medicine, Chiang Mai University, Chiang Mai, 50200, Thailand

**Keywords:** resistance to thyroid hormone, TSH-producing pituitary adenoma, thyroid hormone receptor beta, thyroid hormone receptor mutation

## Abstract

Elevated concentrations of T3 and T4 concomitant with nonsuppressed TSH are found in both TSH-producing tumors and resistance to thyroid hormone beta (RTHβ), posing a diagnostic challenge. We demonstrate here a 54-year-old female who presented with palpitations, goiter, and elevated free T4 with nonsuppressed TSH concentrations (TSH 2.2 mIU/L [normal range, NR 0.27-4.2 mIU/L] and FT4 59.08 pmol/L [NR 12.0-22.0 pmol/L]). Because magnetic resonance imaging revealed a pituitary microadenoma (4 mm), she was diagnosed with TSH-secreting pituitary adenoma and underwent transsphenoidal surgery. Pathological reports showed no tumor cells. Subsequent genetic testing revealed a pathogenic variant in the *THRB* gene resulting in a His435Arg amino acid substitution in the T3 receptor isoform beta 1 (TRβ1), suggestive of RTHβ. In vitro and ex vivo studies revealed that the His435Arg mutated TRβ1 (TRβ1-H435R) completely abolishes the T3-induced transcriptional activation, nuclear receptor corepressor 1 release, steroid receptor coactivator 1 recruitment, and T3-induced thyroid hormone target gene expression, confirming the pathogenicity of this variant. The identification of a pituitary microadenoma in a patient with RTHβ led to a misdiagnosis of a TSH-producing tumor and unnecessary surgery. Genetic testing proved pivotal for an accurate diagnosis, suggesting earlier consideration in similar clinical scenarios.

## Introduction

Resistance to thyroid hormone beta (RTHβ) is caused by pathogenic variants in the *THRB* gene, which encodes thyroid hormone receptor beta isoforms (TRβ) (1). Patients with RTHβ present with inappropriate TSH secretion, characterized by elevated thyroid hormone (TH), T3, and T4, with nonsuppressed TSH concentrations. Recently, Campi et al reported a 12% misdiagnosed rate among 100 subjects with inappropriate TSH secretion, including a patient with RTHβ misdiagnosed with a TSH-producing tumor (2). Individual case reports also highlight this diagnostic challenge (3-6). It is important to distinguish RTHβ from TSH-producing tumors given the differing clinical management (2, 4, 7).

Genomic actions of TH depend on the binding of T3, the biologically active form of TH, to TRs. The crystal structure and in silico model of T3-bound wild-type (WT) TRβ1 protein reveal that the His435 and the Arg282 residues form a His-Arg clamp that plays a crucial role in stabilizing T3 in TRβ ligand-binding pocket (8-10). Pathogenic variants of His435 (ie, His435Leu, His435Gly, and His435Pro) have been previously described in patients with RTHβ (11, 12), confirming the importance of this amino acid residue on T3 binding TRβ function. Recently, a His435Arg pathogenic variant has been reported in 1 family with RTHβ (13). However, extensive functional studies to confirm pathogenicity of this pathogenic variant has never been performed.

Here, we report a 54-year-old woman with RTHβ who was misdiagnosed with a TSH-secreting pituitary adenoma caused by a His435Arg pathogenic variant in the *THRB* gene. Early genetic testing could help avoid unnecessary treatments. In vitro and ex vivo studies also confirm functional impairment of the His435Arg pathogenic variant.

## Case Presentation

**Figure 1.**
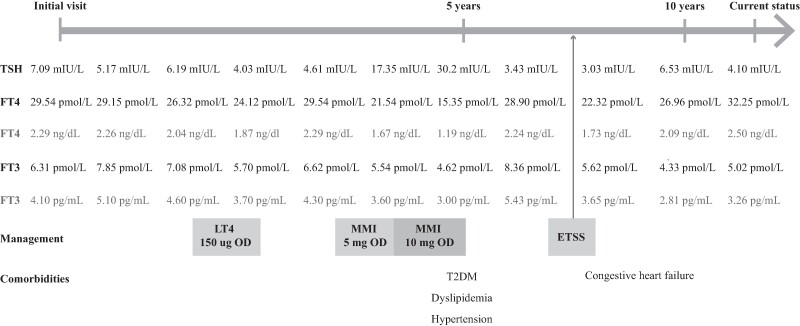
Diagram demonstrates thyroid function tests (TFTs) and treatments of index patient throughout the follow-up period. Levothyroxine (LT4) was given (maximum 150 μg/day) but could not suppress TSH levels. Methimazole (MMI) would normalize free thyroxine (FT4) and free triiodothyronine (FT3) but resulted in elevated TSH levels. TFTs were unchanged after endoscopic transsphenoidal surgery (ETSS). (Reference ranges: TSH 0.27-4.2 mIU/L, FT4 12.0-22.0 pmol/L [0.9-1.7 ng/dL], and FT3 3.13-6.76 pmol/L [2.0-4.4 pg/mL]; unit conversion: TSH mIU/L × 1 = μIU/mL, FT4 pmol/L × 0.0777 = ng/dL, FT3 × 0.651 = pg/mL).

The index case is a 54-year-old female who presented with palpitations and hypertension since the age of 42 years. She was previously healthy with normal growth and development and had no history of mood or attention problems. Electrocardiogram showed paroxysmal atrial fibrillation (AF) with a rapid ventricular response (heart rate 122/minute). She had a slightly diffuse thyroid gland enlargement (estimated thyroid size, 30 g) and no exophthalmos. Ultrasound of the thyroid gland demonstrated a size of the right and left lobe of the thyroid of 2.24 × 2.34 × 4.93 cm and 2.52 × 1.76 × 6.50 cm, respectively. Initial workup to identify causes of AF revealed high free T4 with nonsuppressed TSH (TSH 2.2 mIU/L [normal range, NR 0.27-4.2 mIU/L] and FT4 59.08 pmol/L [4.59 ng/dL; NR 12.0-22.0 pmol/L; 0.93-1.71 ng/dL]). The levels of total and free T3 were not checked at that time. There was no evidence of other pituitary hormone deficiency or excess. A beta-blocker and anticoagulant were prescribed to control her AF. Her heart rate was controlled in the normal range. She was also diagnosed with obesity (body weight 76 kg, body mass index 33 kg/m^2^) and subsequently developed hypertension, congestive heart failure, type 2 diabetes mellitus, and dyslipidemia requiring multiple drugs (diuretics, angiotensin-converting enzyme inhibitor, sulfonylurea, metformin, and simvastatin) treatment (Fig. 1). During the follow-up period, levothyroxine (LT4) was given at a maximum dose of 150 μg/day (2 μg/kg/day), with close monitoring and after controlling the AF, to observe the TSH responses. It was shown that LT4 could not suppress her TSH concentration, suggesting autonomous TSH secretion. She was subsequently treated with methimazole (maximum 10 mg/day), which normalized her FT4 and FT3 but resulted in further elevated TSH concentrations (Fig. 1). Magnetic resonance imaging demonstrated a 4-mm pituitary microadenoma at the left posterior part of the pituitary gland with no significant change in size in 4 years’ follow-up. Serum alpha glycoprotein subunit, TRH stimulation test, and T3 suppression test were not performed because of limited availability of these tests. The thyroid function tests of her younger sister and her son were normal. Because of high TH with nonsuppressed TSH, unchanged TSH levels after exposure to a supraphysiologic dose of LT4, and evidence of a pituitary microadenoma, a TSH-secreting pituitary microadenoma was suspected. Therefore, endoscopic transsphenoidal surgery was performed to remove the suspected pituitary microadenoma. Pathological reports showed multiple pieces of the anterior lobe and a piece of the posterior lobe with no tumor cells. Postoperative magnetic resonance imaging showed no gross residual pituitary microadenoma. After surgery, her thyroid function tests still showed high TH with nonsuppressed TSH, which led to the suspicion of RTHβ.

## Diagnostic Assessment

### THRB Mutation Analysis

**Figure 2.**
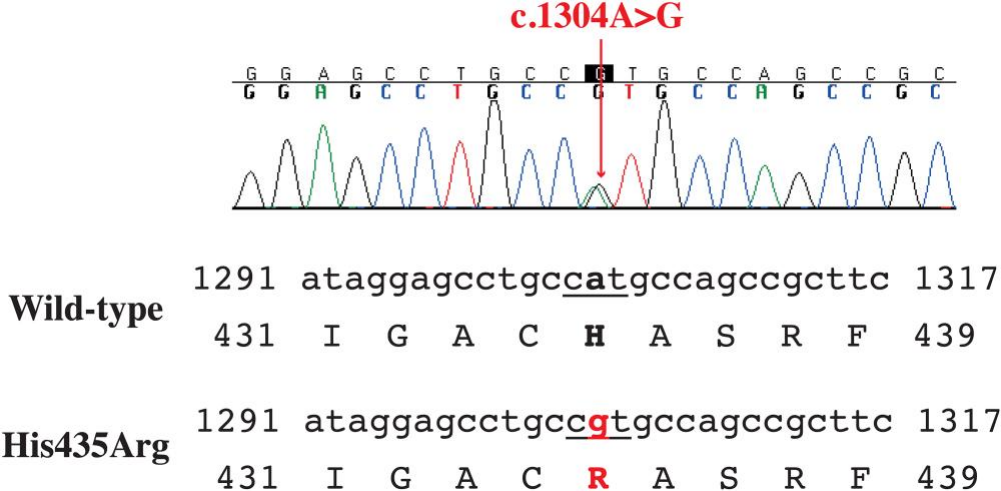
Sequence analysis of exon 10 of *THRB* gene shows a heterozygous missense mutation (c.1304A>G) in index patient, resulting in a His to Arg substitution at codon 435 (His435Arg).

Exons 7-10 of the *THRB* gene of the index patient were sequenced as previously described (9) after obtaining approval from the ethics committee (PED-2565-09214) and informed consent from the patient. A heterozygous missense pathogenic variant at codon 435 resulting in a histidine to arginine substitution (c.1304A>G, p.His435Arg) was identified (Fig. 2).

### Transcriptional Activity of His435Arg Mutated TRβ1 (TRβ1-H435R)

**Figure 3.**
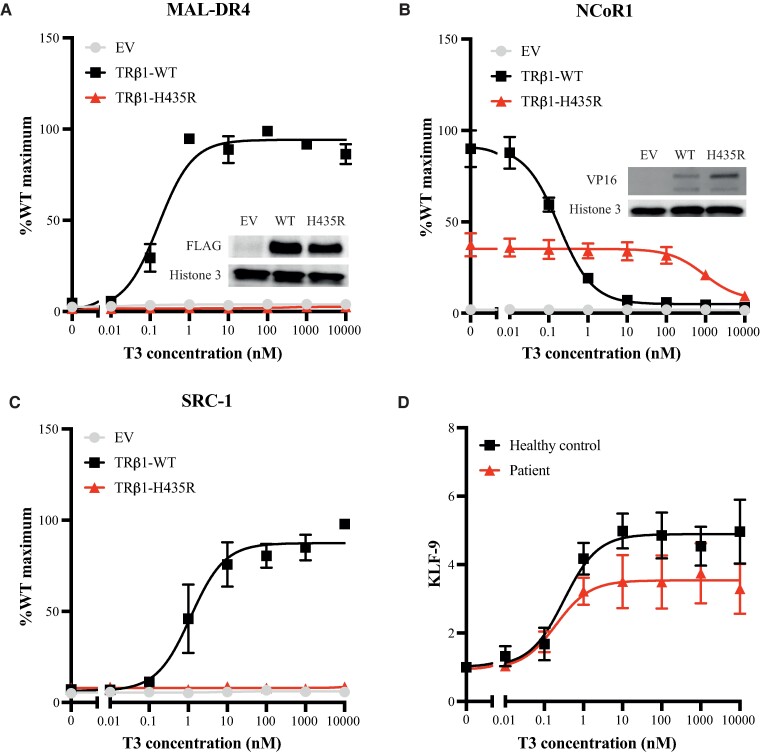
(A) The TRβ1-H435R abolishes all T3-dependent transcriptional activity, as indicated by no activation on DR4-TRE at any of the concentrations tested. (B) T3 induced GAL4-NCoR1 dissociation and (C) GAL4-SRC1 association curves show a severe reduced T3-dependent NCoR1 release and no SRC1 recruitment at any of the concentrations tested. All the transfection experiment data presented as mean ± SEM of 3 independent experiments performed in triplicate. (D) The expression of the T3-responsive gene KLF9 in the fibroblast of the patient, who carries His435Arg heterozygous mutation of the TRβ, and a healthy control was evaluated by quantitative real time PCR using TaqMan probes. Expression levels were normalized to the housekeeping gene GAPDH and quantified using the ddCt method. The KLF9 expression in patient's fibroblasts was lower than that of the healthy control's cells. Data are presented as mean ± SEM of 3 independent experiments performed in duplicate. Immunoblotting confirms the expression of all receptor constructs (insets).

Transcriptional activity of TRβ1-H435R was measured using a reporter construct in which the gene encoding firefly luciferase is under control of a TH response element (MAL-TK-Luc) as previously described (9). T3 induced transcriptional activity for WT TRβ1 in a dose-dependent manner, but not for TRβ1-H435R at any of the concentrations tested (Fig. 3A).

### Cofactor Interactions With TRβ1-H435R

Next, a mammalian 2-hybrid assay as previously described (9) was used to determine the T3-dependent interaction of TRβ1-H435R with the nuclear receptor corepressor 1 (NCoR1) and steroid receptor coactivator 1 (SRC1). Unstimulated TRs bind to NCoR1 to repress gene transcription, whereas T3-stimulated TRs release NCoR1 and recruit SRC1 to promote gene transcription. With NCoR1, the luciferase signal with TRβ1-H435R was 37% of the WT level (Fig. 3B). T3-induced release of NCoR1 from TRβ1 was severely impaired for TRβ1-H435R (IC50 1.01 [0.76-1.35] μM vs 0.18 [0.15-0.21] nM for WT-TRβ1, *P* < .001). In addition, whereas T3 induced SRC1 recruitment for WT-TRβ1 (EC50 1.17 [0.55-2.46] nM), SRC1 did not bind to TRβ1-H435R at any of the T3 concentrations tested (Fig. 3C).

### T3***-***induced TH Target Gene Expression in Patient's Fibroblast

A TaqMan probe-based quantitative PCR assay was performed to quantify expression of the T3-response gene, *KLF9* (*Hs00230918_m1*), which encodes KLF transcription factor 9, in fibroblasts obtained from the patient. The T3-induced expression of the *KLF9* in patient fibroblasts was lower than that of the healthy control's cells, suggesting the dominant-negative effect of this mutation on the WT receptors in the patient's cells (Fig. 3D).

## Treatment

After the diagnosis of RTHβ is confirmed, beta-blocker (Carvedilol 6.25 mg twice daily) was prescribed to control her heart rate. Warfarin, an oral anticoagulant therapy, was also given to prevent thromboembolic events related to her paroxysmal atrial fibrillation. No TH replacement or antithyroid medication is required.

## Outcome and Follow-up

The patient had no symptoms and signs of hypopituitarism after endoscopic transsphenoidal surgery. Her heart rate was controlled at 80 to 90 beats per minute by beta-blocker. Congestive heart failure was controlled with slightly improved cardiac function. Diffuse thyroid gland enlargement was still detected by physical examination and thyroid ultrasonography with no abnormal echogenicity. Her last thyroid function tests revealed high TH with nonsuppressed TSH (Fig. 1).

## Discussion

We report the clinical and biochemical features of an RTHβ patient with a His435Arg pathogenic variant, who was initially misclassified as having a TSH-producing pituitary adenoma. In addition, we characterize the molecular and functional properties of this His435Arg pathogenic variant using different in vitro tests and ex vivo test in patient-derived cells.

Mutations of TRβ result in reduced T3 binding affinity. Mutated TRβ in the pituitary gland resists negative feedback by THs, leading to elevated T3 and T4 levels with nonsuppressed TSH. The clinical presentation of RTHβ varies and depends on tissue-specific TH resistance. Goiter is the main symptom prompting medical advice (14-16). Tachycardia, short stature, and attention deficit disorders may occur because of high THs in tissues where the other types of TR (ie, TRα) predominates, such as the heart, bones, and brain. Most patients are asymptomatic as high TH production compensates for tissue resistance, requiring no treatment. Selective beta-blockers can be considered in patients with tachycardia (1, 14, 15, 17).

Our case reemphasizes the need to have a proper workup for atypical thyroid function tests. The identification of a pituitary microadenoma led to a misdiagnosis of a TSH-producing tumor, resulting in an unnecessary pituitary adenomectomy. Using genetic testing for the identification of mutations in *THRB* gene proved pivotal in achieving an accurate diagnosis. However, it should be noted that mutations in the *THRB* gene cannot be identified in approximately 15% of RTH patients (1).

The differentiation between a TSH-secreting pituitary adenoma and RTHβ can pose a diagnostic challenge. Individuals who have symptoms of thyrotoxicosis, neurological manifestations indicative of optic chiasm compression, and observed co-hypersecretion of additional anterior pituitary hormones, such as prolactin or GH, are more likely to have a TSH-secreting pituitary macroadenoma (1, 2, 18). However, 20% to 30% of patients with TSH-secreting pituitary adenoma are identified as having microadenoma. Within this subgroup, patients may present with milder thyrotoxic and neurological symptoms and might not necessarily demonstrate concomitant pituitary hormone hypersecretion (19, 20), thereby complicating differentiation from RTHβ. The extent of thyroid function abnormalities that are disproportionate to the size of the pituitary adenoma may help distinguish RTHβ from a TSH-secreting tumor. Identifying persistently inappropriate TSH secretion through a historical review of the patient's thyroid function tests, along with similar thyroid function patterns in first-degree relatives, can aid in diagnosing RTHβ (1). Additional tests such as a TRH stimulation test or T3 suppression test along other biochemical markers such as serum alpha glycoprotein subunit and SHGB are important diagnostic tools (2, 18). Limited availability of these tests in many settings is a significant barrier and may lead to misdiagnosis and unnecessary surgery, as demonstrated in our patient. In such case, genetic testing can guide the correct diagnosis. Because liothyronine (T3) was not available in our country, LT4 was administered cautiously after controlling AF in our patient, lacking standard protocol support. This revealed a nonsuppressible TSH and erroneously indicating a TSH-producing tumor. However, administering LT4 to patients with AF is potentially harmful and should not be routinely used to suggest the diagnosis. Although a pituitary adenoma might represent a TSH-producing adenoma, the number of incidentally discovered pituitary adenomas on brain imaging may amount up to 22.5% of healthy individuals (21) and may also be present in patients with RTHβ (2, 7). Nevertheless, cases of coexisting RTHβ with TSH-secreting pituitary adenoma confirmed through histopathological examination have also been rarely documented (22, 23).

**Figure 4.**
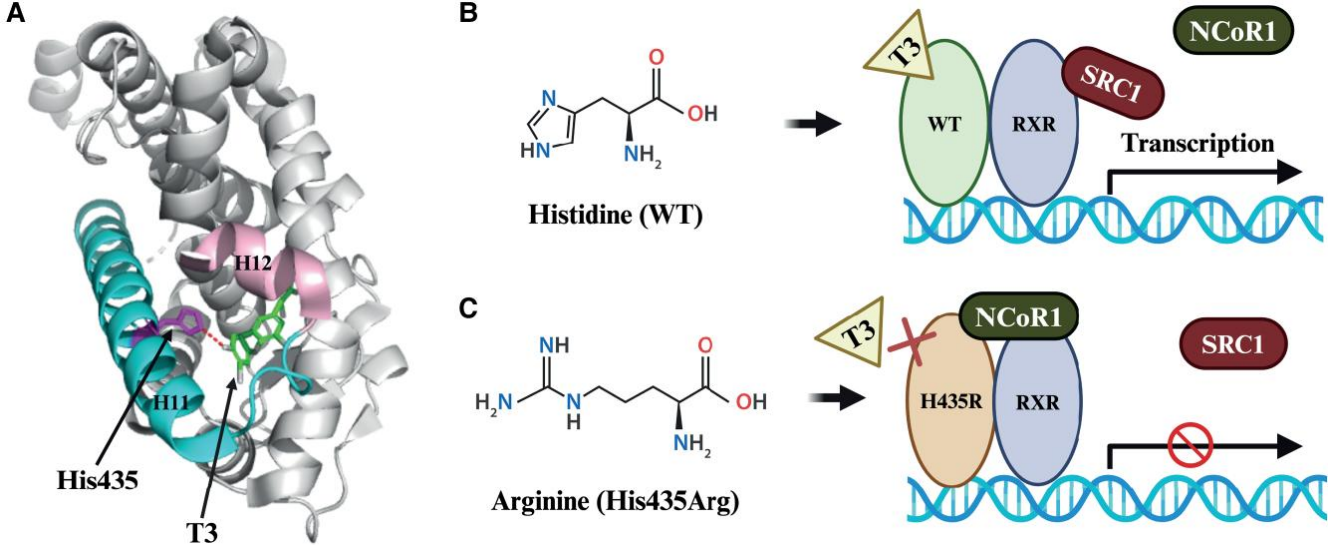
(A) The structure of T3-bound WT TRβ1 (PDB-ID: 3GWS (8)) shows the side chain of His435 and the T3 molecule in the ligand-binding pocket (H, helix). The hydrogen bond between His435 and the phenol hydroxyl group of T3 is illustrated with a dashed line. (B) The structure of the His side chain is depicted. WT TRβ1 containing His435 binds to T3 normally, resulting in heterodimerization with retinoid X receptor (RXR), nuclear receptor corepressor 1 (NCoR1) release, steroid receptor coactivator 1 (SRC1) recruitment, and transcriptional activation. (C) Substituting His with Arg, which has a different side chain structure, leads to low T3 binding affinity, impaired NCoR1 release and SRC1 recruitment, and reduced transcriptional activation. (This figure was created with Biorender.com and the PyMOL Molecular Graphics System, Version 3.0 Shcrödinger, LLC.)

The His435Arg pathogenic variant identified in our patient was previously reported in 1 large family with RTHβ (7 adults and 4 newborn babies) (13). All individuals harboring this mutation exhibited high levels of TH alongside with nonsuppressed TSH concentrations. The clinical manifestations, including tachycardia and symptoms of hyperthyroidism, varied among family members. However, the molecular properties of this pathogenic variant were never characterized. Our in vitro studies showed that the TRβ1-H435R completely abolishes the T3-dependent transcriptional function of TRβ1, which is likely attributable to the strongly reduced affinity of TRβ1-H435R for T3. This is further underscored by the severely impaired T3-dependent NCoR1 release and lack of SRC1 binding, which results in persistent suppression of gene transcription, even in the presence of high concentrations of T3. The effects of the His435Arg mutation on TRβ function are summarized in Fig. 4.

Previous studies report that the inherent binding mode constitutes a “His-Phe switch,” which means that the hydroxyl group of T3, His435 in helix 11 and Phe459 in helix 12 form a stable interaction and represent an inherent binding mode (24). However, TRβ1-H435R breaks His-Phe switch, resulting in insensitivity of the mutant for T3. Recently, a compound named 16 g was designed that can activate the TRβ1-H435R mutant through hydrophobic interactions that replace the His-Phe switch to stabilize helix 12, which results in TRβ1-H435R activation with an EC50 value of 1.34 μM (25). In our in vitro study, we found that the corepressor NCoR1 was released from TRβ1-H435R under supraphysiological concentrations (IC50 value of 1.01 μM). However, this did not result in transcriptional activity. This implies that TRβ1-H435R can still bind T3 and release NCoR1 but in contrast to 16 g cannot recruit SRC1 and activate transcription.

In summary, we present a case of a patient with RTHβ who was initially misdiagnosed with a TSH-producing microadenoma, emphasizing the challenge in diagnosis of these two conditions. Nonnormalized thyroid functions persisted after pituitary surgery, and the identification of the His435Arg pathogenic variant confirmed the diagnosis of RTHβ in this patient. Functional studies on TRβ1-H435R further confirmed the abolished T3-dependent transcriptional activity of this pathogenic variant, underscoring the critical role of His435 in ligand-induced TRβ1 activity.

## Learning Points

- Patients with RTHβ may be incorrectly diagnosed as having a TSH-producing pituitary adenoma because of similarities in thyroid function test results, characterized by elevated TH and nonsuppressed TSH levels.
- Early genetic testing for mutations in the *THRB* gene is crucial to achieving an accurate diagnosis and avoiding unnecessary treatment.
- The histidine to arginine substitution at codon 435 in the TRβ1 isoform results in the reduced ligand-induced transcriptional activity, highlighting the functional importance of this amino acid residue in receptor function.

## Data Availability

Some or all datasets generated during and/or analyzed during the current study are not publicly available but are available from the corresponding author on reasonable request.
